# Production of a Novel Functional Fruit Beverage Consisting of Cornelian Cherry Juice and Probiotic Bacteria

**DOI:** 10.3390/antiox7110163

**Published:** 2018-11-12

**Authors:** Ioanna Mantzourani, Chryssa Nouska, Antonia Terpou, Athanasios Alexopoulos, Eugenia Bezirtzoglou, Mihalis I. Panayiotidis, Alexis Galanis, Stavros Plessas

**Affiliations:** 1 Laboratory of Microbiology, Biotechnology and Hygiene, Faculty of Agriculture Development, Democritus University of Thrace, 68200 Orestiada, Greece; cnouska@agro.duth.gr (C.N.); alexopo@agro.duth.gr (A.A.); empezirt@agro.duth.gr (E.B.); splessas@agro.duth.gr (S.P.); 2 Food Biotechnology Group, Department of Chemistry, University of Patras, GR-26504 Patras, Greece; ant.terpou@gmail.com; 3 Department of Applied Sciences, Faculty of Health & Life Sciences, Ellison Building A516, Northumbria University, Newcastle upon Tyne NE1 8ST, UK; m.panagiotidis@northumbria.ac.uk; 4 Department of Molecular Biology and Genetics, Faculty of Health Sciences, Democritus University of Thrace, Dragana, 68100 Alexandroupolis, Greece; agalanis@mbg.duth.gr

**Keywords:** cornelian cherry juice, *Lactobacillus plantarum* ATCC 14917, fermentation, wheat bran, phenolic content, functional beverage

## Abstract

The present study describes the development of a novel functional beverage through the application of probiotic *Lactobacillus plantarum* ATCC (American Type Culture Collection) 14917 in Cornelian cherry juice fermentation. The probiotic was employed in free and immobilized in a delignified wheat bran carrier (DWB) form. Cornelian cherry juice was fermented for 24 h and then it was stored at 4 °C for 4 weeks. Several parameters were evaluated such as residual sugar, organic acid and alcohol levels, total phenolics content, and cell viability as well as consumers acceptance. Regarding sugar and organic acids analyses, it was proved that the probiotic free or immobilized biocatalyst was effective. The concentration of ethanol was maintained at low levels (0.3–0.9% *v*/*v*). The total phenolic content of fermented Cornelian cherry juice with immobilized cells was recorded in higher levels (214–264 mg GAE/100 mL) for all the cold storage time compared to fermented juice with free cells (165–199 mg GAE/100 mL) and non-fermented juice (135–169 mg GAE/100 mL). Immobilized cells retained their viability in higher levels (9.95 log cfu/mL at the 4th week) compared to free cells (7.36 log cfu/mL at the 4th week). No significant sensory differences were observed among the fermented and the non-fermented samples.

## 1. Introduction

During the last few decades, the concept of food has changed greatly. Functional foods have attracted increasing attention and, as a result, the food industry has turned to the satisfaction of consumers’ demands for more nutritious and safer food products [1]. Based on recent developments, it is anticipated that fermented bio-functional beverages will continue to be a significant category within the functional food market [2,3]. In addition, increasing scientific evidence claims that some foods and food components have beneficial physiological and psychological effects over and above the provision of the basic nutrients. Likewise, the food industry moved to the introduction of functional foods as foods product delivering additional or enhanced benefits over and above their basic nutritional value [4]. The term “functional food” was first introduced around the mid-80s in Japan, which was the first country that stated a specific regulatory approval process for functional foods, while in Europe the interest in functional foods started in the 1990s [5]. However, functional foods are either closely regulated in some countries, or are not even recognized by law in others [6]. For example, in Japan, the term “functional food” was recognized as “foods with specific health claims” approved only under the provision of regulatory processes [7]. Likewise, functional foods are considered one of the most expanding areas of research and innovation in the food field, as suggested by the increasing number of scientific research papers dealing with this topic [1,4,6].

The market of functional foods is characterized by an increasing attention due to their health-promoting attributes, while some researchers have reported that probiotic food products represent approximately 60–70% of functional foods [8]. Probiotic foods are defined as foods containing live microorganisms (mainly bacteria) which, when consumed in adequate amounts, can confer health benefits to the host [9]. In addition, prebiotics are also considered also a significant category of functional foods [10]. Prebiotics are food components that promote the growth of particular bacteria in the large intestine that are beneficial to intestinal health and, at the same time, inhibit the growth of bacteria that are potentially harmful to intestinal health [10]. Even though nowadays dairy fermented products are considered as optimum food carriers of probiotic bacteria [11], many reports have occurred regarding lactose-intolerance causing nausea, bloating, or pain or in some cases allergies to the consumers after dairy product consumption [12,13]. As a result, the food industry is searching for new vehicles in order to deliver the health benefits of probiotics [14].

Lately, various fruit juices have been reported as novel and appropriate mediums for probiotic beverage production due to their content of essential nutrients and general acceptance from all consumers group regardless of age, male, geographic region, etc. [14,15,16,17,18,19,20,21]. Among them Cornelian cherry (*Cornus mas* L.) juice has been scarcely reported as a substrate for the production of probiotic beverages during lactic acid fermentation [16,22,23]. Cornelian cherry grows in Asia and Southern Europe. It is proposed as a medicinal plant since it is currently used in medicine to modify kidney and liver functions for their diuretic and anti-diabetic characteristics [24]. It is a very good source of sugars, organic acids, anthocyanin, phenolic compounds, and ascorbic acid [25]. In addition, a recent research outcome revealed that Cornelian cherry possesses antibacterial and radioprotective properties against several pathogens and exhibits antihistamine, cytotoxic, anti-malarial, and anti-inflammatory effects [26]. On the other hand, the viability of probiotics in fruit juices is of great importance because the critical concentration of viable cells for a probiotic food product is about 10^6^–10^7^ cfu/mL at the time of consumption [27]. Many methods have been studied for the enhancement of the viability of food probiotics, such as cell entrapment or immobilization on various food-grade carriers [28,29,30]. Delignified wheat grain (DWB) is considered a promising carrier for cell immobilization lately since it possesses prebiotic properties due to its composition (50% dietary fibre, 20% protein, 7% ash, 4% lipids) and has been applied successfully in probiotic cheese production [31].

Therefore, the main target of the present study was to produce a new functional low alcoholic beverage through the fermentation of a highly nutritive fruit juice (Cornelian cherry) by the probiotic *Lactobacillus plantarum* ATCC 14917 strain. Likewise, the fermentative ability of the *Lactobacillus plantarum* ATCC 14917 for lactic acid fermentation will be evaluated. A second target is to enhance the viability of probiotic cells of *Lactobacillus plantarum* ATCC 14917 through immobilization [29]. This is the first time that immobilized probiotic cells will be applied for Cornelian cherry juice fermentation. The viability of the strain, the total phenolic content, the consumer acceptance, as well as the concentration of residual sugars, organic acids, and ethanol, were monitored during storage at 4 °C for four weeks. 

## 2. Materials and Methods

### 2.1. Microorganism

The probiotic strain *Lactobacillus plantarum* ATCC 14917 was selected and applied in the fermentations [32]. It was grown under anaerobic conditions at 37 °C for 48 h in de Man, Rogosa, and Sharpe (MRS) broth (Fluka, Buchs, Switzerland). Wet biomass was harvested by centrifugation (Sigma 3K12, Bioblock Scientific, Illkirch Cedex, France) at 5000 rpm for 10 min at 25 °C [29,32]. All media were autoclaved at 120 °C and at 1–1.5 atm for 15 min before use.

### 2.2. Immobilized Biocatalyst Preparation

Wheat bran (*Triticum aestivum* L.) was supplied by a local flour industry (Kepenos Mills, Patras, Greece), and was used as an immobilization carrier of *L. plantarum* cells as described by previous studies [29,31]. In brief, wheat bran was delignified by boiling it in 1% NaOH solution and sterilized by autoclaving at 120 °C, 1.5 atm for 15 min. Immobilization was performed by mixing 5 g of DWB with 1 g of harvested *L. plantarum* ATCC 14917 cell mass (dry weight) in 500 mL MRS broth and incubating at 37 °C for 48 h. The biocatalyst (DWB with naturally entrapped *L. plantarum* ATCC 14917 cells) was washed with sterile 1/4 strength Ringer’s solution for the removal of free cells [31]. The biocatalyst was frozen to −44 °C by a cooling rate of 5 °C min^−1^, and freeze-dried for 48 h at 5–15 mbar and at −45 °C on a FreeZone 4.5 Freeze-Drying System (Labconco, Kansas City, MO, USA). No cryoprotectant was used during the free-drying of the immobilized cells [31]. 

### 2.3. Cornelian Cherry Juice Fermentation

Ripe Cornelian cherry fruits were bought from a local market of Orestiada (Greece). The fresh fruits were carefully selected, washed, the kernel was removed, and it processed into juice by blending for 10 min in a common household blender. The final retentate was added into deionized water (1:1 proportion) and the mixture was submitted to blanching at 90 °C constant temperature for 2 min. The remaining juice was extracted by cloth filtration and was pasteurized at 80 °C for 5 min. The initial value of pH was adjusted to 3.5 through the addition of NaOH 0.1 M, while the initial sugar concentration was 55 g/L. Two grams (dry weight) of free and immobilized respectively *L. plantarum* ATCC 14917 was added in 100 mL of fermentation substrates. The samples were incubated for 24 h at 30 °C and kept for 4 weeks under cold storage (4 °C). The fermentations were carried out in triplicate.

### 2.4. Microbiological Analysis

Samples of 10 mL were taken from the Cornelian cherry juice at various time intervals (days 1, 7, 14, 21 and 28) during fermentation and cold storage. The samples were blended with 90 mL of sterile ¼ strength Ringer’s solution (Merck, Taufkirchen, Germany) and mixed in a stomacher blender and then subjected to serial decimal dilutions in a ¼ strength Ringer’s solution. *L. plantarum* ATCC 14917 cells were recorded on acidified MRS agar (Merck, Taufkirchen, Germany) at 37 °C for 72 h, anaerobically (Anaerobic jar, Anerocult C, Merck, Taufkirchen, Germany). All cell counts were expressed as the log of mean colony forming units (CFU) per mL of Cornelian cherry juice. The results are presented as means of three repetitions plus standard deviations. 

### 2.5. Ethanol and Residual Sugar Analysis

Residual sugar (glucose, fructose, and sucrose) and ethanol concentration were determined with high-performance liquid chromatography by collecting samples at various time intervals (days 1, 7, 14, 21 and 28). Samples were filtered through 0.2-μm microfilters, before injection on a Shimadzu HPLC system (Shimadzu, Kyoto, Japan) consisting of an SCR-101N stainless steel column, an LC-9A pump, a CTO-10A oven set at 60 °C, and a RID-6A refractive index detector. Ultra-pure water obtained by a Milli-Q water purifying system (resistivity 18.2 MΩ cm^−1^) was used as the mobile phase with a flow rate of 0.8 mL/min, and 1-butanol (0.1% *v*/*v*) was used as the internal standard. Ethanol (% *v*/*v*) and residual sugar (g/L) concentrations were calculated using standard curves. All the results are presented as means of at least three repetitions plus standard deviations.

### 2.6. Organic Acid Analysis

The determination of the main produced organic acids (lactic and acetic) was conducted with ion-exchange liquid chromatography. The analysis was carried out on an ion-exchange HPLC Shimadzu system consisting of a Shim-pack ICA1 column, an LC-10AD pump, a CTO-10A oven, and a CDD-6A conductivity detector. A solution of 2.5 mM phthalic acid and 2.4 mM tris (hydroxymethyl) aminomethane (pH 4.0) was used as the mobile phase (1.2 mL/min). The column temperature was 40 °C. The sample dilution was 5% *v*/*v* and the injection volume was 60 μL. Determinations were carried out by means of standard curves.

### 2.7. Total Phenolics 

The total phenolics content was determined by using the Folin-Ciocalteu reagent based on colourimetric reduction [33]. Folin-Ciocalteau (10%, *w*/*v*,) of 1 mL is added to 0.2 mL of prepared Cornelian cherry juice, followed by the addition of 1.2 mL of aqueous Na_2_CO_3_ (7.5%, *w*/*v*). The mixture was left in the dark for 90 min. The absorbance of the blue colour solution was monitored at 760 nm on a UV visible spectrophotometer (Shimadzu, Kyoto, Japan) against a blank (distilled water). Total phenolics concentration (mg/100 mL) of the samples were analyzed in triplicates and extrapolated from a standard curve constructed by using Gallic acid as a standard. Results were expressed as mg Gallic acid equivalents (GAE)/100 mL juice. All measurements were repeated three times.

### 2.8. Consumer Study

A consumer study of the non-fermented and fermented Cornelian cherry juices with *L. plantarum* ATCC 14917 was performed as described previously [34]. Three parameters were evaluated (aroma, taste, and overall quality). The evaluation was carried out by a panel of 30 non-trained laboratory members after the end of the juice fermentation and during storage at 4 °C.

### 2.9. Statistical Analysis

The data obtained from, physicochemical characteristics, total phenolics content, cell viability and sensorial analysis of the non-fermented and fermented Cornelian cherry juice were analysed for their mean differences with the Analysis of Variance (ANOVA) procedure followed by Duncan’s post hoc multiple range test to extract the specific differences between the various treatments. The analysis was performed by using IMB SPSS v20 (IBM Corp, Armonk, NY, USA.) at an alpha level of 5%.

## 3. Results and Discussion

### 3.1. Cell Survival

Changes in cell viability of *L. plantarum* ATCC 14917 cells in Cornelian cherry juice that were free (FC) or immobilized (IC) after the fermentation of 24 h and cold storage (4 °C) for 4 weeks are presented in Table 1. Even though the viability of FC and IC cells decreased during the cold storage, all the achieved viability values were over the critical limit in all cases (10^6^ to 10^7^ cfu/mL), which is required for probiotic products [27]. Specifically, the viability of FC decreased about 4 folds (7.36 log cfu/mL at the 4th week of cold storage), while the viability of IC decreased approximately 1-fold (9.95 log cfu/mL at the 4th week of cold storage). It seems that the adjustment of the Cornelian cherry juice pH value to 3.5 from 2.8, which was the pH value of the freshly prepared juice, supported cell viability. This slight increase was made in order to ameliorate the survival of *L. plantarum* ATCC 14917 as other researchers have proposed [22] and fulfil the criteria of the food industry that requires minimal processing treatments [35]. On the other hand, there are reports claiming that the type of probiotic or inhibitory compounds can affect cell viability on fruit juices [36]. For instance, there is a report demonstrating that phenolic compounds present in fruit juices may exert possible prebiotic effects on probiotics increasing their viability [37]. It is also noteworthy that *L. plantarum* ATCC 14917 displays a high acid resistance ability [38] while cell immobilization further enhanced cell viability. After the 1st week of cold storage, the viability of IC was statistically higher compared to the viability of FC. This may be credited to the protective effect of the prebiotic immobilisation substrate, as other researchers have demonstrated the employment of DWB as a carrier of lactic acid bacteria for fermented dairy products production [31,39]. Furthermore, there are reports in the literature claiming that immobilization clearly protects cells from the acidic environment of the juices and leads to a limited viability loss [40,41].

### 3.2. Ethanol, Organic Acids, and Residual Sugar Concentrations

The results (residual sugar, lactic and acetic acid and ethanol) obtained through fermentation of Cornelian cherry juice with free (FC) and immobilized (IC) in DWB *L. plantarum* ATCC 14917 cells are presented in Table 2. In general, residual sugar levels were decreased while the levels of organic acids were increased, indicating the efficiency of free and immobilized *L. plantarum* ATCC 14917 for lactic acid fermentation of Cornelian cherry juice. This outcome is in accordance with a previous research dealing with the fermentation of Cornelian cherry with free *L. plantarum* ATCC 14917 cells [16]. However, in this case, the fermentation temperature was set to 37 °C and a lower initial amount of the microorganism was employed (approximately 7 log cfu/mL) compared to the present study where the fermentation temperature was set to 30 °C and the respective amount was 11.15 log cfu/mL. Residual sugar concentration was reduced by approximately 62% (20.9 g/L), in the case of FC and 66% (18.9 g/L) in the case of IC at the 4th week of cold storage respectively (statistically significant). It is noteworthy, that the achieved residual sugar concentrations were statistically significant for all the periods of time studied in the case of IC compared to FC. Likewise, it is obvious that *L. plantarum* ATCC 14917 cells were protected in the immobilization support and were more efficient during fermentation compared to FC. The concentration of lactic acid was increased statistically significantly every week reaching its maximum value at the 4th week of cold storage (97.8 mg/100 mL, in the case of FC and 148.5 mg/100 mL in the case of IC). In addition, during all the 4 weeks of cold storage, the application of IC led to statistically higher values compared to IC. Acetic acid was determined for all the time periods examined except for the first 24 h of fermentation for both FC and IC. Afterwards, it was produced continuously, reaching its maximum value at the last week (8.2 mg/100 mL in the case of FC and 15.4 mg/100 mL in the case of IC). The ethanol concentration was increased from 0.3% *v*/*v* at the first 24 h of fermentation to 0.7% *v*/*v* and 0.9% *v*/*v*, with regards to FC and IC, respectively, at the 4th week of cold storage. Between FC and IC, no statistically significant differences were observed.

### 3.3. Total Phenolics Content (TPC) 

Fermented Cornelian cherry juice by FC and IC was also examined regarding total phenolics content (TPC) after 24 h of fermentation and for each week of cold storage (Table 3). For comparison reasons, non-fermented Cornelian cherry juice (NFC) was applied as the control. Initial total phenolics content of freshly prepared Cornelian cherry juice was about 175.15 ± 10.19 mg GAE/100 mL. In general, the application of *L. plantarum* ATCC 14917 in free or immobilized form for the fermentation of Cornelian cherry led to higher values of TPC compared to NFC and, especially at the last three weeks of cold storage, the differences were obviously significant (Table 2). Fermented Cornelian cherry juice by FC possessed higher TPC from the control juice and especially after the 2nd week of cold storage the differences were statistically significant. However, the best results were achieved in the case of IC application. In particular, in all the time periods examined, the recorded TPC of Cornelian cherry juice fermented by IC was statistically significantly higher compared to the TPC values of NFP and Cornelian cherry juice fermented by FC (Table 2). Specifically, the TPC of Cornelian cherry juice fermented by IC reached its higher values in the first week of cold storage (257.20 mg GAE/100 mL) and for all the other 3 weeks (264.71 mg GAE/100 mL at the 2nd, 258.84 mg GAE/100 mL at the 3rd week, and 249.61 mg GAE/100 mL at the 4th week). The recorded amelioration of TPC is likely to be attributed to the lactic acid fermentation as other researchers have demonstrated [34,42,43]. Many studies regarding the fermentation of fruit juices with lactic acid bacteria have revealed an increase in TPC [20,21,37]. In particular, fermentation of fig juice by *L. plantarum* caused the microbial enzyme release producing high levels of flavonoids, tannin, alkaloids, and phenylpropanoid [43,44]. Likewise, it seems that lactic acid fermentation stimulates the conversion of the simple phenolic compounds and the phenolic compound depolymerization with the high molecular weight [45].

The present outcome is more encouraging compared to a recent study conducted with the application of various probiotic strains in Cornelian cherry fermentation [22]. In that case, phenolic content was slightly decreased in contrast to the present study that was increased. On the other hand, immobilization may act as a protective agent for the *L. plantarum* ATCC 14917 cells enhancing its fermentative activity leading to higher rates of fermentation [29,31] and the degradation of conjugated forms of phenolics as other researchers have pointed [46].

### 3.4. Consumer Study

The consumers’ study was performed by non-trained testers (consumers) to evaluate the produced non-fermented and fermented juices with FC and IC in terms of aroma, taste, and overall quality (preference). The outcome showed that there were no statistically significant differences among all the examined juices at the same time of fermentation and cold storage (Table 4). Even though there are reports claiming that consumers seem to prefer natural juices than fermented ones with probiotic bacteria [47], there was no off-flavour in the fermented Cornelian cherry juice with the FC and IC of *L. plantarum* ATCC 14917, in agreement with a previous study [22].

## 4. Conclusions

The outcome of the present work showed that the probiotic *Lactobacillus plantarum* ATCC 14917, immobilized on DWB, can be successfully used for a low alcohol functional Cornelian cherry beverage production. The enhanced viability of *L. plantarum* ATCC 14917 during cold storage was observed due to the prebiotic effect of DWB. The fermented juice after fermentation and during the whole cold storage period had a high total phenolics content. In addition, there was no off-flavour in the fermented Cornelian cherry juice according to the preliminary sensory evaluation conducted. Likewise, the proposed product containing high amounts of probiotic bacteria and significant nutritional properties due to Cornelian cherry juice could be a good alternative to specific consumer groups seeking natural and high nutritive beverages.

## Figures and Tables

**Table 1:** The viability of free (FC) and immobilized (IC) *Lactobacillus plantarum* ATCC 14917 cells in the fermented Cornelian cherry juices after fermentation (24 h in 30 °C) and over 4 weeks of storage at 4 °C.

Time	Cell Counts (log cfu/mL)	
	FC	IC
0 h	11.15 ± 0.4 ^A,a^	11.15 ± 0.3 ^A,a^
24 h	10.28 ± 0.2 ^A,b^	10.47 ± 0.2 ^A,b^
Week 1	9.21 ± 0.3 ^B,c^	10.41 ± 0.3 ^A,b^
Week 2	9.03 ± 0.2 ^B,c^	10.49 ± 0.1 ^A,b^
Week 3	7.86 ± 0.3 ^B,d^	10.08 ± 0.1 ^A,c^
Week 4	7.36 ± 0.2 ^B,d^	9.95 ± 0.1 ^A,c^

Different superscript letters within the rows (A–B) at the same time period of storage, as well as within the columns (a, b, c, and d), indicates statistically significant differences (2 WAY ANOVA, Duncan’s multiple range test, *p* < 0.05).

**Table 2:** The analysis of sugars, organic acids, and ethanol in Cornelian cherry juice fermented by free (FC) and immobilized *Lactobacillus plantarum* ATCC 14917 (IC).

Parameter	Time	*Lactobacillus plantarum* ATCC 14917	
		FC	IC
**Sugars** **(g/l)**	24 h	51.5 ± 0.3 ^A,a^	50.4 ± 0.3 ^B,a^
	Week 1	47.3 ± 0.1 ^A,b^	42.3 ± 0.3 ^B,b^
	Week 2	37.2 ± 0.4 ^A,c^	33.1 ± 0.3 ^B,c^
	Week 3	24.2 ± 0.4 ^A,d^	21.8 ± 0.3 ^B,d^
	Week 4	20.9 ± 0.5 ^A,e^	18.9 ± 0.3 ^B,e^
**Lactic Acid** **(mg/100 mL)**	24 h	11.2 ± 0.2 ^B,e^	24.2 ± 0.1 ^A,e^
	Week 1	39.8 ± 0.2 ^B,d^	41.4 ± 0.1 ^A,d^
	Week 2	58.4 ± 0.2 ^B,c^	76.9 ± 0.2 ^A,c^
	Week 3	81.1 ± 0.1 ^B,b^	94.8 ± 0.3 ^A,b^
	Week 4	97.8 ± 0.3 ^B,a^	148.5 ± 0.4 ^A,a^
**Acetic Acid** **(mg/100 mL)**	24 h	ND	ND
	Week 1	1.2 ± 0.2 ^B,d^	3.4 ± 0.1 ^A,d^
	Week 2	3.9 ± 0.2 ^B,c^	7.6 ± 0.2 ^A,c^
	Week 3	6.4 ± 0.2 ^Bb^	9.2 ± 0.2 ^A,b^
	Week 4	8.2 ± 0.2 ^B,a^	15.4 ± 0.3 ^A,a^
**Ethanol** **(% *v*/*v*)**	24 h	0.3 ± 0.1 ^A,b^	0.3 ± 0.1 ^A,c^
	Week 1	0.3 ± 0.1 ^A,b^	0.3 ± 0.1 ^A,c^
	Week 2	0.4 ± 0.1 ^A,b^	0.6 ± 0.1 ^A,b^
	Week 3	0.4 ± 0.1 ^A,b^	0.6 ± 0.1 ^A,b^
	Week 4	0.7 ± 0.1 ^A,a^	0.9 ± 0.1 ^A,a^

Different superscript letters within the rows (A–B) at the same time period of storage, as well as within the columns (a, b, c, and d) for each parameter examined indicates the statistically significant differences (2 WAY ANOVA, Duncan’s multiple range test, *p* < 0.05).

**Table 3:** The determination of the total phenolics content of non-fermented (NFC) and fermented Cornelian cherry juice with free (FC) and immobilized *Lactobacillus plantarum* ATCC 14917 (IC) in the first 24 h at 30 °C and during storage at 4 °C for 4 weeks.

Time	Total Phenolics Content (mg GAE/100 mL)		
	NFC	FC	IC
**24 h**	169.32 ± 12.23 ^B,a^	165.02 ± 10.62 ^B,b^	214.41 ± 10.56 ^A,b^
**Week 1**	161.32 ± 11.30 ^B,a^	184.45 ± 10.23 ^B,a^	257.20 ± 17.11 ^A,a^
**Week 2**	136.28 ± 10.08 ^C,b^	199.72 ± 14.51 ^B,a^	264.71 ± 16.08 ^A,a^
**Week 3**	136.40 ± 10.53 ^C,b^	198.38 ± 19.06 ^B,a^	258.84 ± 13.24 ^A,a^
**Week 4**	135.19 ± 11.08 ^C,b^	195.02 ± 14.75 ^B,a^	249.61 ± 14.34 ^A,a^

Different superscript letters within the rows (A, B and C) at the same time period of storage, as well as within the columns (a, b, c, and d) for each parameter examined, indicates statistically significant differences (2 WAY ANOVA, Duncan’s multiple range test, *p* < 0.05).

**Table 4:** A consumer study regarding aroma, taste, and overall quality of non-fermented fermented (NFC), fermented Cornelian cherry juice with free (FC) and immobilized *Lactobacillus plantarum* ATCC 14917 (IC) in the first 24 h at 30 °C and during storage at 4 °C for 4 weeks.

Storage Time	Substrate	Aroma	Taste	Overall Quality
24 h	NFC	8.5 ± 0.1 ^a^	9.0 ± 0.2 ^a^	9.1 ± 0.1 ^a^
	FC	8.6 ± 0.2 ^a^	9.1 ± 0.2 ^a^	8.9 ± 0.1 ^a^
	IC	8.5 ± 0.1 ^a^	9.0 ± 0.2 ^a^	9.0 ± 0.2 ^a^
Week 1	NFC	7.8 ± 0.2 ^a^	7.8 ± 0.1 ^a^	7.7 ± 0.2 ^a^
	FC	7.9 ± 0.1 ^a^	7.9 ± 0.2 ^a^	7.8 ± 0.2 ^a^
	IC	7.7 ± 0.1 ^a^	7.6 ± 0.1 ^a^	7.7 ± 0.1 ^a^
Week 2	NFC	7.0 ± 0.1 ^a^	6.9 ± 0.1 ^a^	7.0 ± 0.2 ^a^
	FC	7.1 ± 0.1 ^a^	6.8 ± 0.1 ^a^	7.1 ± 0.2 ^a^
	IC	7.0 ± 0.1 ^a^	6.9 ± 0.1 ^a^	7.0 ± 0.1 ^a^
Week 3	NFC	6.5 ± 0.1 ^a^	6.5 ± 0.2 ^a^	6.5 ± 0.2 ^a^
	FC	6.6 ± 0.1 ^a^	6.6 ± 0.1 ^a^	6.5 ± 0.2 ^a^
	IC	6.4 ± 0.1 ^a^	6.5 ± 0.2 ^a^	6.5 ± 0.1 ^a^
Week 4	NFC	6.3 ± 0.3 ^a^	6.1 ± 0.2 ^a^	6.1 ± 0.3 ^a^
	FC	6.2 ± 0.2 ^a^	6.1 ± 0.3 ^a^	6.3 ± 0.2 ^a^
	IC	6.3 ± 0.2 ^a^	6.0 ± 0.4 ^a^	5.9 ± 0.2 ^a^

Different superscript letters in a column at the same time of period of storage indicates statistically significant differences (ANOVA, Duncan’s multiple range test, *p* < 0.05).
